# Mechanistic investigations of polyaza[7]helicene in photoredox and energy transfer catalysis

**DOI:** 10.3762/bjoc.20.106

**Published:** 2024-05-28

**Authors:** Johannes Rocker, Till J B Zähringer, Matthias Schmitz, Till Opatz, Christoph Kerzig

**Affiliations:** 1 Department of Chemistry Johannes Gutenberg University, Duesbergweg 10–14, 55128 Mainz, Germany

**Keywords:** energy transfer, laser spectroscopy, organocatalyst, photoredox, time-resolved spectroscopy

## Abstract

Organic photocatalysts frequently possess dual singlet and triplet photoreactivity and a thorough photochemical characterization is essential for efficient light-driven applications. In this article, the mode of action of a polyazahelicene catalyst (Aza-H) was investigated using laser flash photolysis (LFP). The study revealed that the chromophore can function as a singlet-state photoredox catalyst in the sulfonylation/arylation of styrenes and as a triplet sensitizer in energy transfer catalysis. The singlet lifetime is sufficiently long to exploit the exceptional excited state reduction potential for the activation of 4-cyanopyridine. Photoinduced electron transfer generating the radical cation was directly observed confirming the previously proposed mechanism of a three-component reaction. Several steps of the photoredox cycle were investigated separately, providing deep insights into the complex mechanism. The triplet-excited Aza-H, which was studied with quantitative LFP, is formed with a quantum yield of 0.34. The pronounced triplet formation was exploited for the isomerization reaction of (*E*)-stilbene to the *Z*-isomer and the cyclization of cinnamyl chloride. Catalyst degradation mainly occurs through the long-lived Aza-H triplet (28 µs), but the photostability is greatly increased when the triplet efficiently reacts in a catalytic cycle such that turnover numbers exceeding 4400 are achievable with this organocatalyst.

## Introduction

**Scheme 1:**
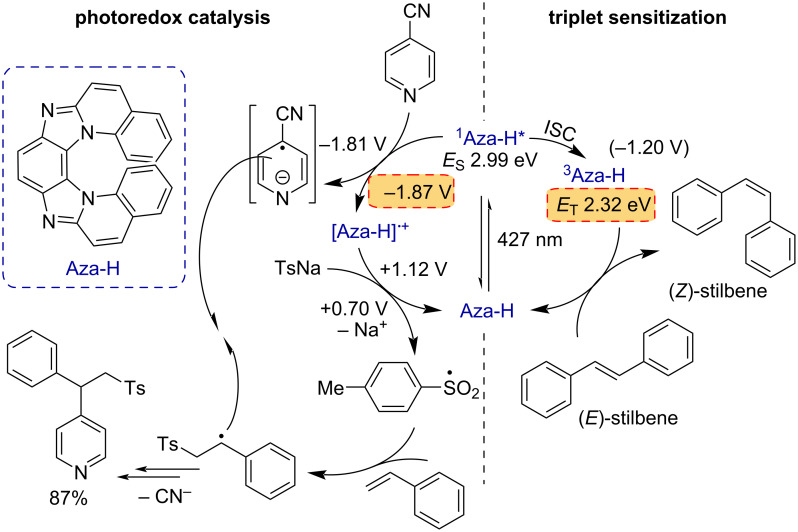
Left: Reaction mechanism of the 3-CR with Aza-H as the photocatalyst. Potentials are given vs SCE. Right: Isomerization of stilbene using Aza-H as triplet sensitizer.

The emergence of photoredox chemistry in recent years was pioneered by the introduction of photocatalysts (PC) based on metals such as Ru and Ir [1–6]. However, due to the high cost and limited availability of precious metals, organic photocatalysts have become a focal point of academic and industrial research [7–15]. Most of the established organic catalysts (acridinium salts [16–19], cyanoarenes [8,20–22], quinones [23–24], etc.) [10,25] are cationic or electron-deficient and tend to act as excited state oxidants in a reductive quenching cycle. Only recently, more reducing catalyst classes have been investigated, including second-generation cyanoarenes [8], arylamines [26], phenothiazines, phenazines and phenoxazines [9,27–28], which can act as excited state reductants comparable to precious metal-based photoredox catalysts. Singlet-excited organic chromophores often have short lifetimes, limiting their use in collision-based electron/energy transfer reactions unless high substrate concentrations are used [29–30]. In contrast, triplet states populated via intersystem crossing (ISC) have much longer lifetimes and can efficiently react at lower substrate concentrations [30]. Prominent examples include: (i) carbonyl compounds such as benzophenone [31–32] and thioxanthone [33–35], (ii) TADF emitters with small singlet-triplet energy splitting [8,36], (iii) substitution with heavy atoms such as sulphur [37] and halides [38] for fast ISC, or a combination of the above-mentioned methods [39]. However, the longer lifetime of the triplet state usually comes at the expense of a lower energetic driving force. Helicenes in particular show an increased ISC rate compared to planar molecules, which is attributed to an enhanced spin-orbit coupling that is sensitive to their degree of non-planarity [40], where the helical structure allows for mixing between the singlet and triplet π,π*-states [41–42]. In addition, the insertion of heteroatoms such as nitrogen further enhances ISC due to a spin-orbit coupling between n,π*-states and π,π*-states [40], as stated by El Sayed’s rule [43]. However, helicenes have not been broadly considered as potential photocatalysts or sensitizers or their applications were unsuccessful [44]. Recently, one of our groups exploited the highly reducing polyaza[7]helicene (Aza-H, see Scheme 1 for its structure) for sulfonylation/arylation three-component reactions (3-CRs) and it has been shown that substituting the helicene core with functional groups is a viable approach to fine-tune the photochemical properties [45–46], as has been demonstrated for other organic and metal-based chromophores.

Our study focused on investigating the reaction mechanism of the recently reported sulfonylation/arylation [45–46] reaction using laser flash photolysis (LFP). LFP is a powerful spectroscopic tool in photocatalysis that allows us not only to distinguish between energy and electron transfer but also to detect transient triplet states and radicals, yielding clear-cut evidence for the proposed reaction mechanism [47–57]. We found that quenching of the singlet-excited Aza-H by 4-cyanopyridine is the main pathway for the 3-CR, while the triplet state of our catalyst, which is formed with a quantum yield as high as 0.34, is essentially non-reactive under our conditions. Cyanopyridine- and sulfinate-derived radicals are produced in equal concentrations in the catalytic cycle, suggesting that radical coupling is indeed the final reaction step to give the stable sulfonylation/arylation product. The triplet of Aza-H with its relatively high formation quantum yield and an energy of 2.32 eV can be used for isomerizing photoswitches like stilbene and for the cyclization of cinnamyl chloride. Our goal was not only to clarify the reaction pathway but also to provide a clear method for distinguishing between singlet and triplet reactivity of the Aza-H photocatalyst through spectroscopic measurements. The careful characterization of the versatile Aza-H photochemistry might contribute to the development of a new class of photoactive catalysts that can compete with traditional metal complexes and well-known organic chromophores (as listed above).

## Results and Discussion

A thorough understanding of the operation principles of a photocatalyst is essential for the development of improved catalysts and efficient photoreactions. To this end, the photochemical properties of photocatalysts such as metal complexes and organic chromophores and their roles in catalytic cycles have been extensively studied leading to numerous findings and novel reaction pathways [55,58–61]. Lately, polyazahelicenes have gained some attention by synthetic groups [62–66]. However, this chromophore class is underexplored concerning its photochemical reactivity. The main reaction class catalyzed by excited-state polyazahelicene Aza-H so far is the redox-neutral addition of sulfinates and cyanopyridines, under elimination of cyanide, to styrenes in a three-component reaction. The proposed mechanism was derived from the redox potentials of Aza-H and the substrates and initial steady-state fluorescence quenching experiments (Scheme 1, left), but detailed mechanistic insights and direct evidence of the transient radical ions could not be obtained yet [45].

**Figure 1:**
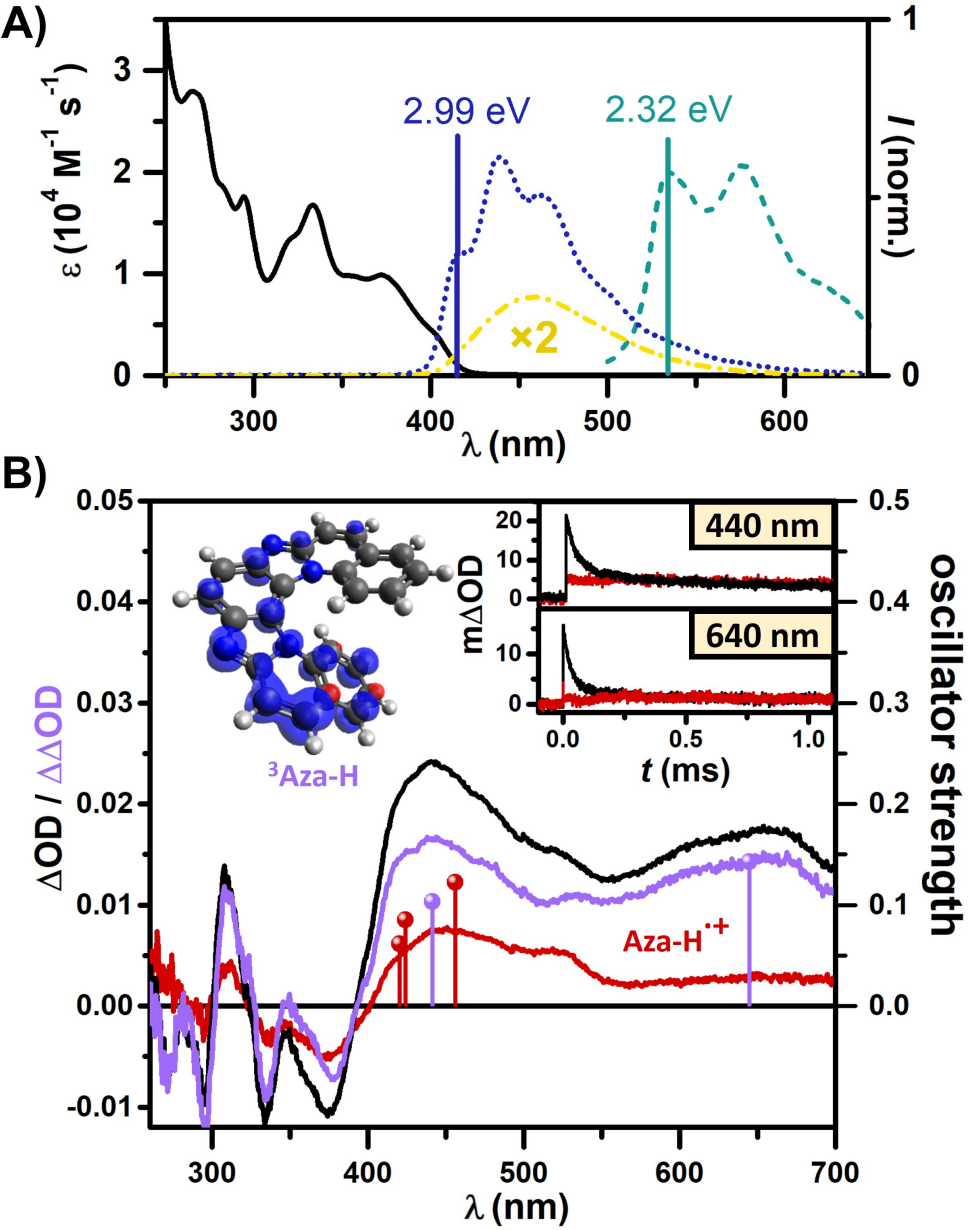
A) Room-temperature absorption (black) and emission (yellow) spectra of Aza-H recorded in MeCN/H_2_O (9:1), and fluorescence (blue) and phosphorescence (green) spectra measured at 77 K in MeOH/EtOH (4:1). B) Transient absorption spectra of 25 µM (initial ground-state absorbance at the laser wavelength, 0.2) Aza-H in Ar-saturated (black) and air-saturated (red) MeCN/H_2_O (9:1) recorded after 250 ns and the difference spectrum of both (purple) upon excitation with 355 nm laser pulses. Spin density of ^3^Aza-H and the predicted electronic transitions in the visible region (transitions with oscillator strengths
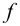
below 0.05 are not shown) of ^3^Aza-H (purple) and Aza-H^•+^ (red). Inset: Kinetic measurements at 440 nm and 640 nm.

Figure 1A illustrates the absorption and emission spectra of Aza-H in MeCN/H_2_O (9:1). The low reduction potential of singlet-excited Aza-H (PC^•+^/PC* = –1.87 V vs SCE) led us to propose that the singlet-excited photocatalyst is oxidized by 4-cyanopyridine (4CP) (4CP/4CP^•−^ = −1.81 V vs SCE) as the first step in this multicomponent reaction (Scheme 1, left). The oxidized photocatalyst (PC^•+^/PC = +1.12 V vs SCE) is then regenerated by an electron transfer from sodium *p*-toluenesulfinate (TsNa) (Ts^•^/Ts^−^ = +0.70 V vs SCE) closing the photocatalytic cycle [45]. Unlike other prominent photocatalysts, little to nothing is known about the reactivity, the photophysical properties or the radical ions of polyazahelicenes. The moderate fluorescence quantum yield of 48% (in MeCN) [46] implies that a non-emissive triplet-excited state (at room temperature) could also be generated via intersystem crossing, potentially initiating electron transfer reaction sequences. 77 K emission measurements were thus carried out and they showed short-lived fluorescence and long-lived emission (phosphorescence) that was assigned to the triplet state of Aza-H with an energy of 2.32 eV (Figure 1A). Quantum mechanical computations of the Aza-H triplet state yielded a triplet energy of 2.17 eV (adiabatic), which is in good agreement with the experimental value (see Supporting Information File 1 for further information). Consequently, triplet reactivity cannot be excluded, given that photoinduced electron transfer reactions with excited singlet states or singlet radical pairs frequently suffer from low cage escape quantum yields [67–68].

Aza-H exhibits a relatively long singlet lifetime of 9.6 ns (see Figure 4E), which is sufficient for diffusion-controlled energy or electron transfer at moderate substrate concentrations. Transient absorption (TA) spectra of an Ar-saturated Aza-H solution were recorded with an LFP setup using 355 nm pulses (20 mJ) of ≈5 ns duration (see Supporting Information File 1 for experimental details). A delay of 250 ns was used between excitation and detection, which is sufficiently long to avoid the detection of the singlet-excited state (Figure 1B, black). Two broad TA bands peaking at 441 nm and 640 nm were observed that we assign to ^3^Aza-H. However, on longer time scales the shape of the spectrum changes significantly (Figure S4, Supporting Information File 1) indicating that more than one absorbing species is generated. Measurements on an air-saturated solution under otherwise identical conditions gave a completely different spectrum (Figure 1B, red) and we assume that the triplet state of Aza-H is almost fully quenched by oxygen at the selected delay, which is supported by additional kinetic measurements (see below). The second transient species is identified as the ionization product resulting from a consecutive two-photon absorption at the selected high laser intensity [69–70], yielding the corresponding long-lived radical cation with second-order decay that is typical for oxidized/reduced species, and a solvated electron as by-product. The latter is in equilibrium with the dimeric solvent radical anion in MeCN, it weakly absorbs in our detection window and this species is rather short-lived compared to the triplet as well as the radical cation [71]. The oscillator strengths for the electronic transitions of ^3^Aza-H (purple) and the radical cation Aza-H^•+^ (red) were computed using TD-DFT (see Supporting Information File 1 for details). These computational results, which are included in Figure 1B, align well with the experimentally observed absorption bands in the visible region, supporting our assignment.

The observed behavior of the Aza-H transient species in Ar-saturated/air-saturated solution is also reflected in the kinetic traces at 440 nm and 640 nm (Figure 1B, inset). Following the decay of the transient triplet species (³Aza-H), essentially the same signal is reached irrespective of the presence of oxygen or argon in the solution. The contribution of the radical cation to the TA signal at 640 nm is only minor, which allows us to estimate a triplet lifetime of ≈28 µs at this detection wavelength. Based on our observations, an isolated triplet spectrum can be obtained from the spectral difference between the black and the red spectrum in Figure 1B, as shown in the purple spectrum displayed in Figure 1B (compare to Figure 3B, where the generated radical cation is fully quenched by TsNa).

**Figure 2:**
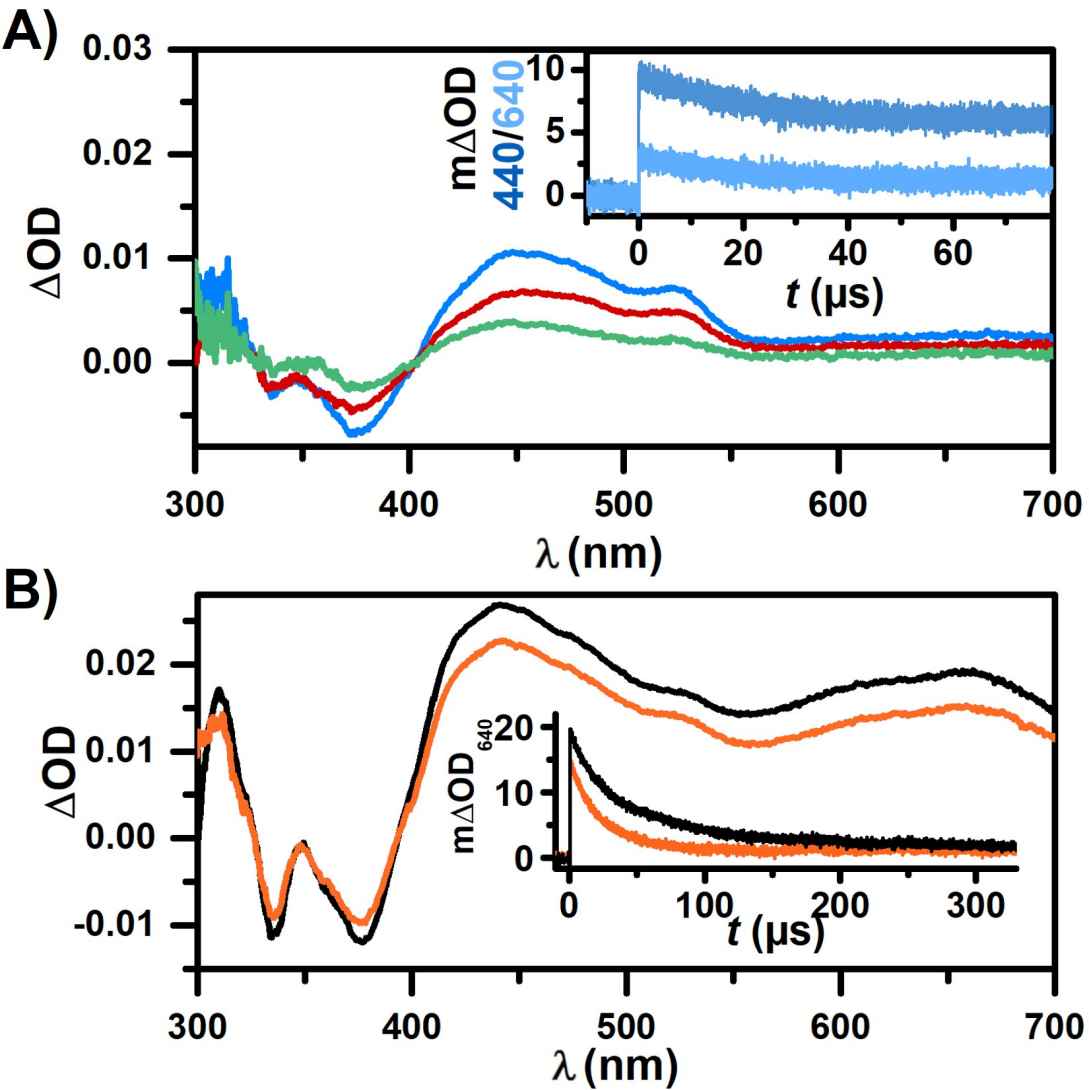
Mechanistic LFP experiments of 25 µM Aza-H with 4CP in MeCN/H_2_O (9:1) after 355 nm laser pulses. A) TA spectra recorded after 250 ns of solely Aza-H in air-saturated solution (red), in Ar-saturated solution with 200 mM 4CP (blue) and the difference spectrum of both (green). Inset: Kinetic traces of Aza-H with 200 mM 4CP at 440 nm and 640 nm. B) TA spectra recorded after 250 ns of Aza-H in Ar-saturated solution in the absence (black) and presence (orange) of 5 mM 4CP. Inset: Kinetic traces at 640 nm.

Since all substrate compounds in the 3-CR mixture (4CP, TsNa and α-methylstyrene) cause fluorescence quenching, albeit to different degrees (see Supporting Information File 1), we recorded post-quenching TA spectra with Aza-H and the different substrates. Adding 200 mM 4CP to a deoxygenated solution of Aza-H quenches the fluorescence almost completely (η > 93%). The post-quenching TA spectrum (250 ns delay) strongly correlates with what we identified as the radical cation, Aza-H^•+^, which is in perfect agreement with our proposed photoinduced electron transfer step, while no triplet-excited Aza-H is observed (Figure 2A, blue spectrum). Since the direct ionization of Aza-H also contributes to this spectrum, we recorded a spectrum of only Aza-H in air-saturated solution (red spectrum) and used it to separate the signal that only stems from the oxidative quenching step (green spectrum). This correction likely overestimates the contribution of the two-photon process to the radical cation signal in the presence of 4CP, considering that quenching competes with ionization already during the laser pulse. Kinetic measurements of the radical cation species show multiexponential decay, probably due to recombination of the radical cation and radical anion (Figure 2A, inset). Despite the reported absorption maximum for 4CP^•−^ at 398 nm [72], we were unable to detect this species, as emphasized by Figure S5 and the discussion in Supporting Information File 1. Similarly, our attempts to identify 4CP^•−^ in an analogous experiment that employed Ir(ppy)_3_ as a well-characterized reference photoreductant were also unsuccessful [73–74], probably due to an overall small extinction coefficient of the radical anion (Figure S6 in Supporting Information File 1 and the corresponding discussion). To investigate the triplet reactivity of Aza-H, a lower concentration of 4CP (5 mM) was used, quenching the singlet excited photocatalyst only partially (η ≈ 25%) and allowing triplet-excited Aza-H to be generated (Figure 2B). Kinetic traces of the triplet state show minor quenching upon the addition of 4CP (Figure 2B, inset), while the initial triplet signal is reduced as expected. However, considering the rather long triplet lifetime (in comparison to the singlet-excited state lifetime), the quenching rate constant is roughly four orders of magnitude lower than singlet quenching (*k*_q_ ≈ 10^5^ M^−1^ s^−1^). This is in good agreement with the reported high triplet energy of 4CP [75] at 3.08 eV compared to 2.32 eV of ^3^Aza-H and the lower reduction potential provided by the Aza-H triplet (−1.20 V vs SCE).

**Figure 3:**
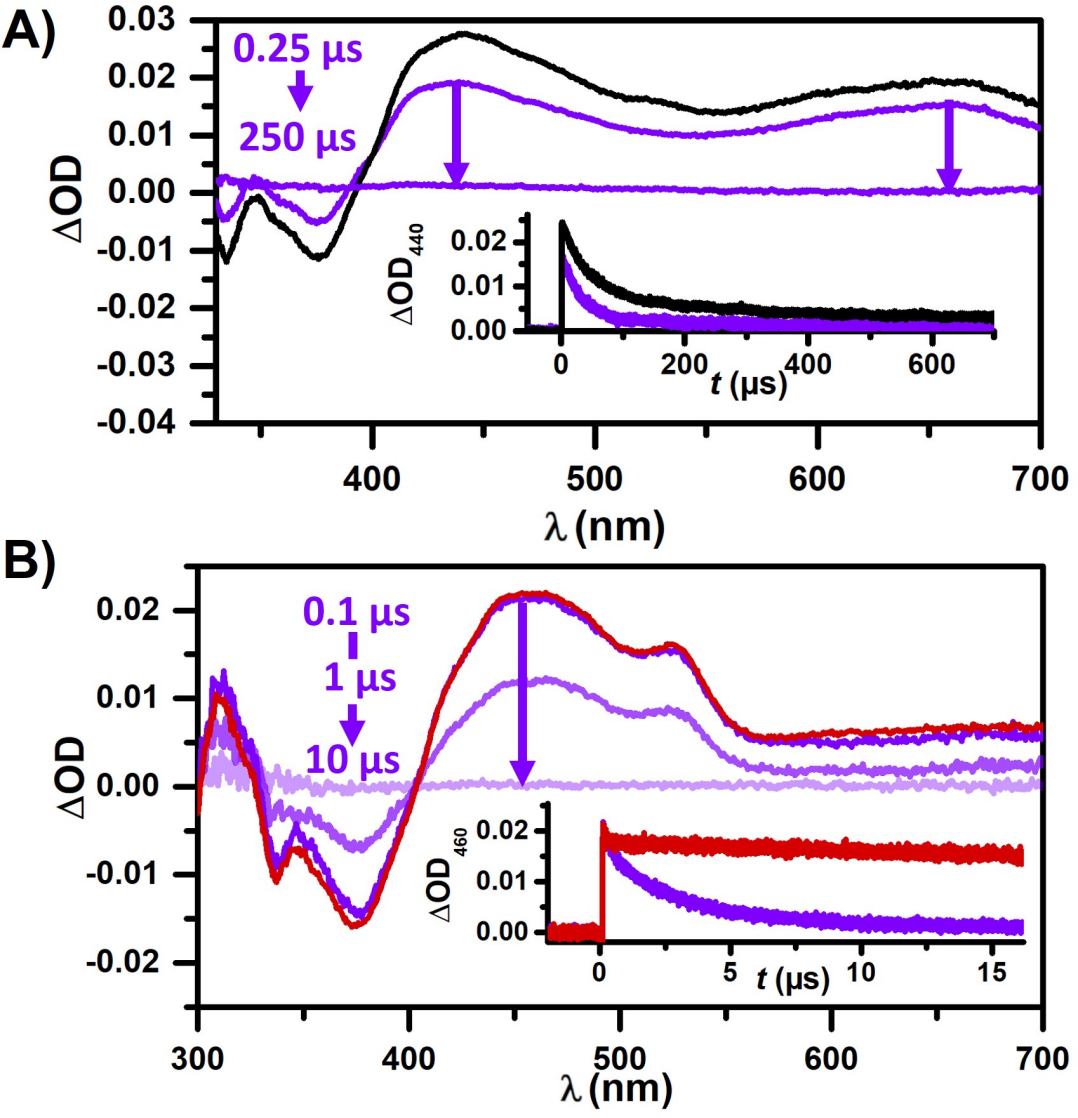
Mechanistic investigations of Aza-H with TsNa by LFP studies. A) Transient absorption measurements of 25 µM Aza-H in the absence of TsNa recorded after 0.25 µs (black) and presence of 50 mM TsNa recorded after 0.25 µs and 250 µs (purple) in deaerated MeCN/H_2_O (9:1). Inset: Kinetic measurements at 440 nm. B) TA spectra of the radical cation of Aza-H (aerated MeCN/H_2_O (9:1) solution) in the absence of TsNa recorded after 0.1 µs (red) and in the presence of TsNa after 0.1 µs, 1 µs and 10 µs (purple). Inset: Kinetic traces at 460 nm.

Hence, we concluded that triplet quenching by 4CP plays a negligible role at typical substrate concentrations (>100 mM) used for lab-scale irradiation experiments. Following the proposed mechanism, the catalyst is then regenerated by an electron transfer from TsNa. However, we also observed minor fluorescence quenching of the photocatalysts by TsNa (see Figure S8, Supporting Information File 1), which prompted us to conduct additional LFP experiments to eliminate this as the primary reaction pathway (Figure 3A). Unlike quenching by 4CP, the addition of TsNa only shows signals that we assigned to the triplet spectrum of Aza-H in Figure 1B. Although the singlet-excited photocatalyst is quenched by TsNa, indicated by the reduced triplet formation of Aza-H, our conclusion is that this quenching is unproductive as no new photoproducts are observed. Interestingly, in contrast to all previous measurements, no signal of the Aza-H radical cation generated through two-photon absorption is detected. This can be easily rationalized as the radical cation is most likely rapidly quenched by TsNa (compare the proposed reaction mechanism regenerating the oxidized catalyst). That reasoning is consistent with kinetic traces at 440 nm that show the isolated decay of triplet Aza-H when TsNa is present, while the longer-lived signatures of Aza-H^•+^ are only visible in the absence of TsNa (Figure 3A, inset). In order to analyse the thermal electron transfer, we prepared an aerated solution of Aza-H with a lower concentration of TsNa (200 µM). We presumed this would enable us to exclusively monitor Aza-H^•+^ quenching by TsNa, since any triplet Aza-H would be deactivated by oxygen (see above). The ionization efficiency of Aza-H exhibits a quadratic dependence on the laser intensity. Consequently, focussing the laser on a narrow excitation spot on the sample ensured a significant generation of Aza-H^•+^ while minimizing the concentration of triplet-excited Aza-H. Initially, the same amount of radical cation is detected after the addition of TsNa, but the Aza-H^•+^ signal at 460 nm decays much faster and reaches the baseline after only 10 µs (Figure 3B). Kinetic measurements yielded a rate constant as high as ≈10^9^ M^−1^ s^−1^ for the reaction between Aza-H^•+^ and TsNa (Figure 3B, inset), which implies that potential side reactions cannot compete. The TA spectra clearly reach baseline level after Aza-H^•+^ quenching by TsNa, indicating that the catalyst is regenerated (ground-state bleach recovery below 400 nm in Figure 3A and 3B) by that reaction, which is in line with the proposed thermal electron transfer. However, both the sulfonyl radical and 4CP^•−^ do not absorb in our detection window, hampering further kinetic and mechanistic investigations. Nevertheless, both radicals must be formed as quenching by-products based on our spectroscopic studies, either in the photoinduced or in the subsequent and rapid thermal electron transfer. For stoichiometry reasons, these radicals are formed in equal concentrations in the catalytic cycle and, therefore, the proposed radical recombination to yield the final product (with intervening addition of the sulfonyl radical to excess styrene, see Scheme 1) can be regarded as most-likely reaction pathway. Furthermore, we carried out p*K* value determinations for the protonation of Aza-H, both in its ground state and in the excited singlet state (see Figure S10 in Supporting Information File 1 and the corresponding section for details). Based on our studies in aqueous ethanol, Aza-H is a weak photobase but protonation is seen to be unimportant in neutral and alkaline solutions. However, these studies might be important for explaining reactivity changes when Aza-H is employed in photoreactions requiring or releasing Brønsted acids. During the 3-CR experiment, we observed a decomposition of the catalyst that occurred faster than in the absence of any additives (see studies on inherent photostability presented below). Our observations also revealed that while the styrene does not significantly quench the singlet state of Aza-H, it quenches the triplet state, suggesting that the formation of triplet-excited styrene via energy transfer is feasible (see Figure S7, Supporting Information File 1) [76]. A reaction course plot in the absence of 4CP and TsNa revealed rapid decomposition of the photocatalyst, presumably in a [2 + 2]-photocycloaddition with the styrene component as observed in mass spectrometry (see Figure S17 and Figure S18 in Supporting Information File 1 for details).

**Figure 4:**
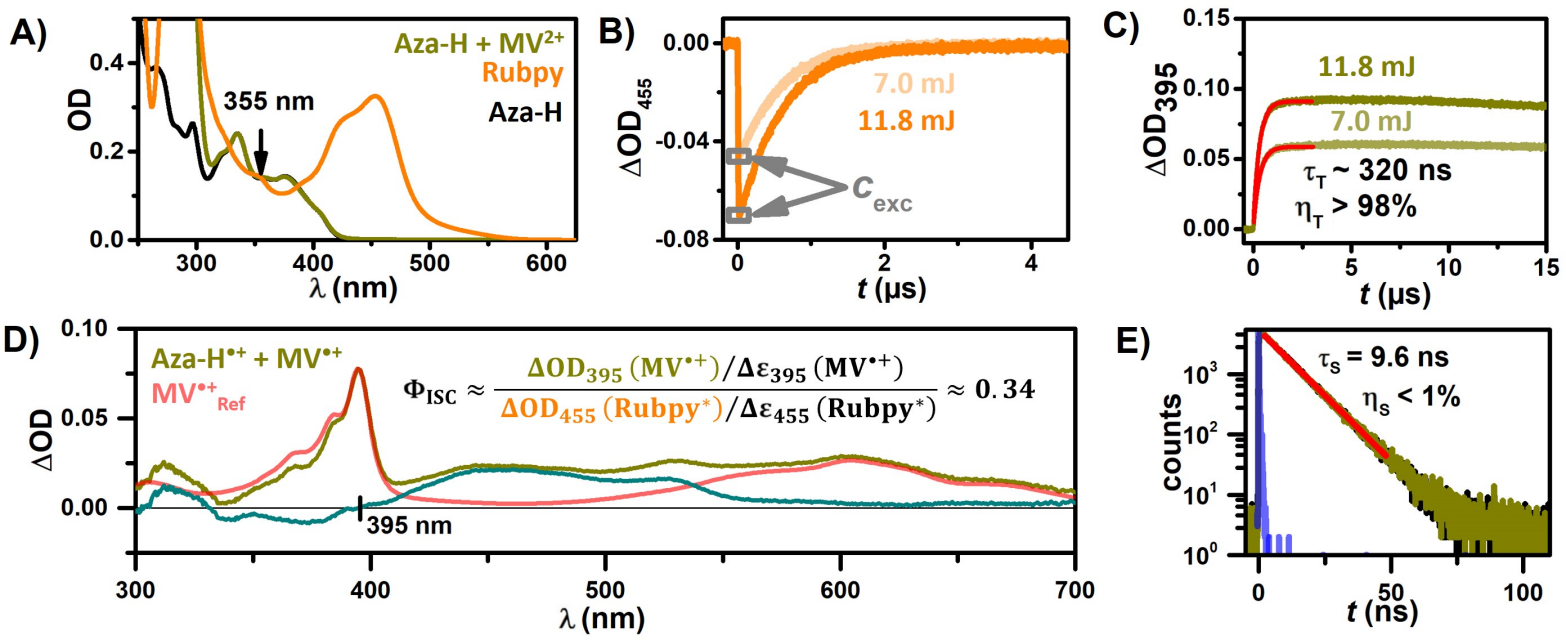
Data sets employed for the calculation Φ_ISC_ of Aza-H based on the ground state bleach of Rubpy as the actinometry reference signal following excitation by 355 nm laser pulses with energies of 7.0 mJ and 11.8 mJ. Aza-H and MV^2+^ were prepared in deaerated MeCN and Rubpy in deaerated H_2_O. A) Absorption spectra of Rubpy, Aza-H, and Aza-H with 0.3 mM MV^2+^. B) and C) Transient absorption measurements of Rubpy at 455 nm and Aza-H with MV^2+^ at 395 nm. D) Absorption spectrum of MV^•+^ obtained from Ref [77], transient absorption spectrum of Aza-H with 0.3 mM MV^2+^ and the difference spectrum of both spectra (cyan). E) Emission lifetime of Aza-H in the presence (dark yellow) and absence (black) of 0.3 mM MV^2+^.

Consequently, the degradation of Aza-H is more efficient at the end of the reaction when incomplete deactivation of the excited singlet state leads to the (more efficient) formation of the triplet state. The occurrence of triplet reactivity of the catalyst as an undesirable side reaction highlights the importance of investigating the triplet state properties and this also initiated our search for a productive use of ³Aza-H. The moderate fluorescence quantum yield of 0.48 indicates that pronounced triplet state formation might take place. Quantitative LFP measurements were performed to determine the ISC quantum yield, Φ_ISC_, using the well-studied photosensitizer [Ru(bpy)_3_]Cl_2_ (Rubpy) as reference for actinometry [78–80]. This approach involved employing identical excitation conditions at 355 nm (Figure 4A) and monitoring the GSB of Rubpy with a literature-reported difference extinction coefficient, Δ*ε*_455_(Rubpy*) = −10100 M^−1^ cm^−1^ [37,79], following excitation (Figure 4B). 0.3 mM methyl viologen (MV^2+^) were added to Aza-H to quantitatively quench the triplet-excited photocatalyst in an electron transfer reaction yielding Aza-H^•+^ and MV^•+^, which are clearly observed in the TA spectrum (Figure 4D). For the Φ_ISC_ determination described below, we assume a cage escape quantum yield of 100%, which is reasonable for the photoinduced electron transfer between a heavy atom-free sensitizer-quencher pair in the triplet manifold [67–68,81]. Quenching of the short-lived singlet excited Aza-H at our low quencher concentration was excluded by lifetime-based quenching experiments (Figure 4E). By observing the kinetic traces at the isosbestic point of Aza-H^•+^ (395 nm), we were able to exclusively track the formation of MV^•+^ (Figure 4C). To rule out the formation of any other products apart from Aza-H^•+^, we utilized a reference spectrum of MV^•+^ obtained through spectroelectrochemistry [77]. The difference spectrum depicted in Figure 4D closely resembles the Aza-H radical cation spectrum already obtained through photoionization and singlet state quenching (see above), substantiating the reliability of our procedure. Taking the literature-known extinction coefficient of the methyl viologen radical cation Δ*ε*_395_(MV^•+^) ≈ 39000 M^−1^ cm^−1^ [82] to calculate its concentration and the initial Aza-H excited-state concentration obtained through Rubpy* actinometry allows us to determine the ISC quantum yield. This procedure was conducted at two excitation intensities: 7.0 mJ and 11.8 mJ (see Supporting Information File 1 for details) yielding an ISC quantum yield of 0.34. The mild excitation energies also ensured that the formation of directly ionized Aza-H was negligible for the evaluation of the ISC quantum yield.

**Figure 5:**
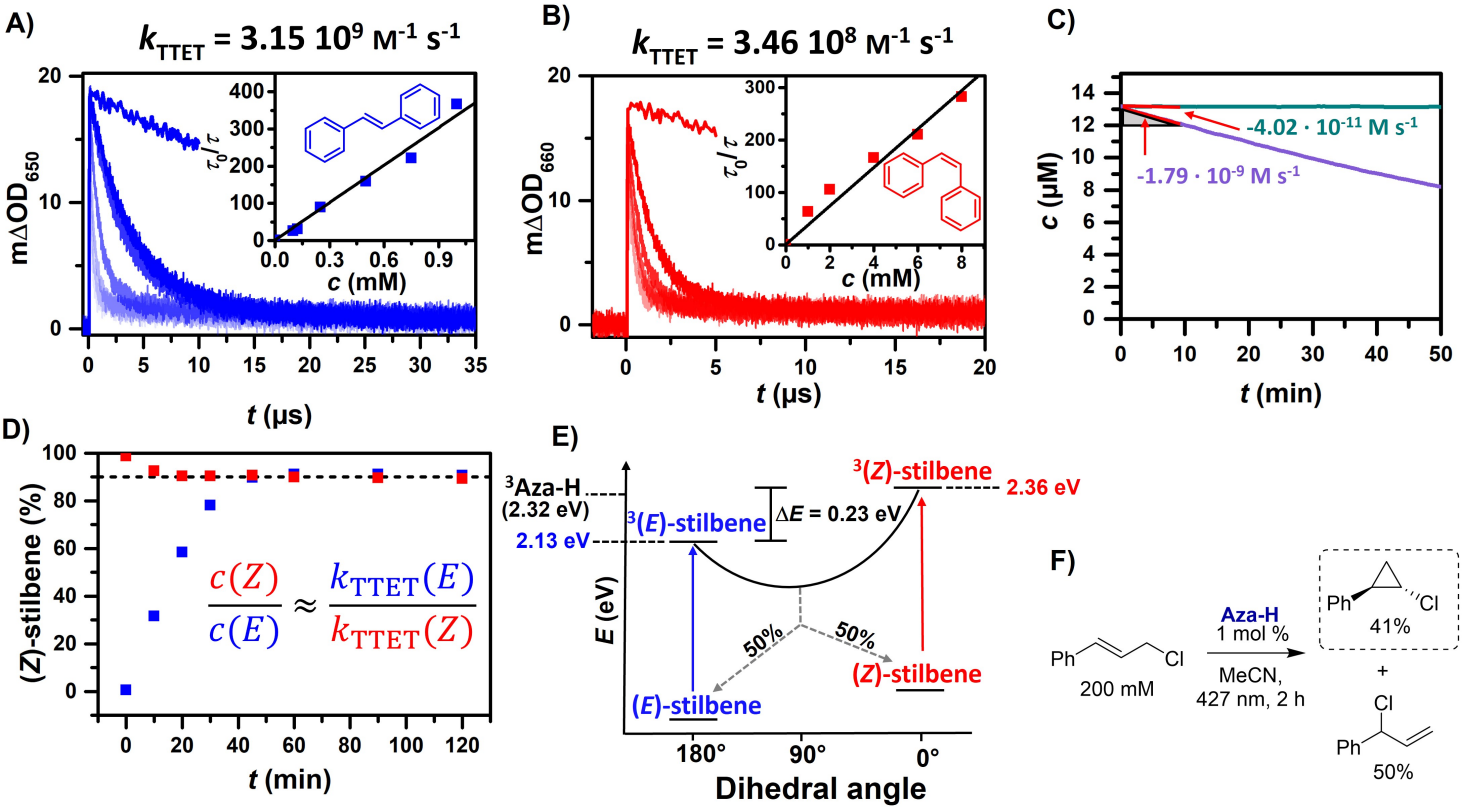
Stilbene isomerization and additional energy transfer experiments. A) and B) Triplet quenching experiments of Aza-H with increasing (*E*)-stilbene and (*Z*)-stilbene concentrations. Insets: Corresponding Stern–Volmer plots. C) Photostability measurements of Aza-H in deaerated MeCN in the absence (purple) and presence of 10 mM (*E*)-stilbene (green). A 390 nm LED from Kessil set to 50% intensity (distance 6 cm) was used as the excitation light source. D) Irradiation experiments of (*E*)-stilbene (blue) and (*Z*)-stilbene (red) with 2 mol % Aza-H employing a 427 nm LED as light source. E) Schematic energy diagram of triplet sensitized *E*/*Z* isomerization of stilbene. F) Reaction scheme of the cyclization of cinnamyl chloride using Aza-H as the triplet sensitizer. See Supporting Information File 1 for details.

With a promising ISC quantum yield of 0.34 and a triplet energy of approximately 2.32 eV, the triplet-triplet energy transfer (TTET) to (*E*)-stilbene (*E*_T_ = 2.13 eV) [76] should be feasible, while an energy transfer to (*Z*)-stilbene (*E*_T_ = 2.36 eV) [76] is energetically uphill and therefore much slower. Triplet quenching experiments of Aza-H with increasing (*Z*)-stilbene and (*E*)-stilbene concentrations were performed (Figure 5A and B). We obtained quenching efficiencies and rate constants that are in line with our measured triplet energy of Aza-H.

Irradiating a 50 mM solution of either (*E*)-stilbene or (*Z*)-stilbene and Aza-H (2 mol %) in MeCN (Figure S19, Supporting Information File 1) reaches an equilibrium at an *Z*/*E*-ratio of 91:9 (Figure 5D). Moreover, the *Z*/*E*-ratio is perfectly reflected by the ratio of the triplet energy transfer rates, which is in line with reported similar efficiencies for the formation of both isomers starting from the perpendicular triplet state (Figure 5E) [35,83].

In contrast to styrene substrates, mass spectrometry shows no addition products of catalyst and stilbene. Hence, the photocatalyst is not consumed by side reactions. Photostability measurements have also demonstrated that the inclusion of a triplet quencher like stilbene significantly enhances the longevity of the Aza-H chromophores, outperforming other well-known organic and metal-based photocatalysts such as 4CzIPN, riboflavin and [Ru(bpy)_3_](PF_6_)_2_ (Figure 5C and Supporting Information File 1). A remarkable turnover number of 4440 was achieved when 1 M stilbene was used (see Supporting Information File 1 for more information). Furthermore, the photocatalyst was utilized in a photosensitized cyclization reaction of cinnamyl chloride [84]. This photoreaction was originally established by Xu et al., but its success was limited to costly Ir-based photocatalysts. Lifetime-based quenching experiments of ^3^Aza-H with increasing cinnamyl chloride concentration revealed an energy transfer rate of 10^6^ M^−1^ s^−1^ (Figure S9, Supporting Information File 1). Although this rate is four orders of magnitude slower than the diffusion limit in MeCN, the long triplet lifetime ensures a quenching efficiency of 85% when a concentration of 200 mM cinnamyl chloride is used. Exposed to 427 nm irradiation with 1 mol % Aza-H 41% of the chlorocyclopropane and 50% of the branched allylic chloride was produced, which can be recycled to the cinnamyl chloride as demonstrated recently [84].

## Conclusion

The experiments presented in this paper have demonstrated the wealth of information accessible by laser spectroscopic studies with a novel polyazahelicene photocatalyst. Its excited singlet state is the key species in a three-component sulfonylation/arylation reaction, whereas the unexplored triplet state is responsible for the decomposition of the photocatalyst. Photoinduced radical cation formation and thermal regeneration could be unambiguously observed, providing deep mechanistic insights into the underlying reaction mechanism. On the other hand, the relatively high triplet formation quantum yield of Aza-H along with its triplet energy on the order of 2.3 eV permit efficient and metal-free reactions via energy transfer catalysis, as shown for the photosensitized isomerization of stilbene and cinnamyl chloride. We believe that our findings pave the way for a broader usage of the inherently chiral polyazahelicene photocatalyst class, both in photoredox and energy transfer catalysis.

## Supporting Information

File 1 General information, detailed experimental procedures, additional spectroscopic data, quantum-mechanical calculations, photostability and photoisomerization experiments.

## Data Availability

All experimental data sets supporting the findings of this study are available in the main article and/or the supporting information. The data sets shown in the main paper and DFT output files are accessible via the JGU library and the homepage of the corresponding author.
