# Supplementary material for: Mechanistic investigations of polyaza[7]helicene in photoredox and energy transfer catalysis
Source: Beilstein J Org Chem. 2024 May 28;20:1236–45. doi: 10.3762/bjoc.20.106 (PMC11181280; doi:10.3762/bjoc.20.106)
Supplement: File 1 — General information, detailed experimental procedures, additional spectroscopic data, quantum-mechanical calculations, photostability and photoisomerization experiments. [file Beilstein_J_Org_Chem-20-1236-s001.pdf]

BEILSTEIN JOURNAL OF ORGANIC CHEMISTRY

Supporting Information

for

Mechanistic investigations of polyaza[7]helicene in photoredox and energy transfer catalysis

Johannes Rocker, Till J. B. Zähringer, Matthias Schmitz, Till Opatz and Christoph Kerzig

Beilstein J. Org. Chem. **2024,** *20,* 1236–1245. doi:10.3762/bjoc.20.106

General information, detailed experimental procedures, additional spectroscopic data, quantum-mechanical calculations, photostability and photoisomerization experiments

License and Terms: This is a supporting information file under the terms of the Creative Commons Attribution License (https://creativecommons.org/licenses/by/4.0). Please note that the reuse, redistribution and reproduction in particular requires that the author(s) and source are credited and that individual graphics may be subject to special legal provisions.
The license is subject to the *Beilstein Journal of Organic Chemistry* terms and conditions: (https://www.beilstein-journals.org/bjoc/terms)

Contents

I. General information S1

II. Quantum-mechanical computations S4

III. Additional mechanistic and kinetic data S5

IV. Photostability measurements S10

V. Additional information for the isomerization of stilbene and cinnamyl chloride S13

VI. References S14

I. General information

Solvents, reagents and reaction setup

All solvents and reagents were obtained from commercial suppliers and used without further purification unless otherwise stated (4-cyanopyridine, 98%, Sigma-Aldrich; riboflavin, >99%, Sigma-Aldrich; (*Z*)-stilbene, 95%, Thermo Scientific; (*E*)-stilbene, 98%, TCI; MeCN, >99%, VWR Chemicals; $[Ru(bpy)_3]Cl_2$, 99.95%, Sigma-Aldrich; cinnamyl chloride, 95%, Thermo Scientific; *fac*-$Ir(ppy)_3$, 99%, Sigma-Aldrich; *N*,*N*′-dimethyl-4,4′-bipyridinium dichloride (methyl viologen), 98%, Sigma-Aldrich; ammonium hexafluorophosphate, 98%, abcr; sodium *p*-toluenesulfinate (TsNa), 95%, Sigma-Aldrich; α-methylstyrene, >99%, Sigma-Aldrich).

α-Methylstyrene was passed through basic Al_2O_3 to remove the stabilizer. 4-Cyanopyridine was sublimed before use. The azahelicene catalyst (Aza-H) was prepared in two steps according to the previously described procedure [1]. The photocatalyst 4CzIPN was synthesized analogous to the method described in Ref [2]. The hexafluorophosphate salts of $[Ru(bpy)_3]Cl_2$ and *N*,*N*′-dimethyl-4,4′-bipyridinium dichloride have been prepared via precipitation from water upon addition of ammonium hexafluorophosphate and thorough washing with water.

Unless mentioned otherwise, argon from Nippon Gases (5.0) was used for removing dissolved oxygen before all experiments related to optical spectroscopy. Aqueous solutions were prepared using ultrapure Millipor Milli-Q water with a specific resistance of 18.2 MΩ cm.

Mass spectra and HPLC

Reversed phase-HPLC measurements and reaction controls were performed on a 1260-series HPLC-system (Agilent) equipped with an integrated diode array detector and coupled with a single quadrupole LC/MSD with ESI source. A C18-column (Ascentis Express C18, 3 cm × 2.1 mm × 2.7 µm) and a flow rate of 0.7 mL/min with a gradient of 10–90% MeCN/H_2O + 0.1% during 10 min were used for routine analysis.
High-resolution mass spectra were recorded on a 6545 QTOF-LC/MS (Agilent).

Gas chromatography

Gas chromatography was performed on an 8890 GC (Agilent) with split-detection on a 5977 single quadrupole MSD and an FID using helium as carrier gas. The stationary phase used was a HP 5MS UI GC column (Agilent, 30 m × 0.25 mm, film thickness 0.25 µm) and the temperature program as follows: injector temp. 250 °C, transfer line temp. 250 °C, MS source temp. 230 °C, quadrupole temp. 150 °C oven temperature 2 min @ 40 °C → 50 °C/min to 320 °C → 7.4 min @ 320 °C.

Steady-state measurements

UV–vis absorption spectra were recorded on a Thermo Scientific Evolution 201 UV-Visible Spectrophotometer and a Perkin Elmer LAMBDA 365 UV-Vis spectrophotometer at room temperature (295 ± 3 K) in 10 mm quartz cuvettes.

Fluorescence spectra were recorded on an FP-8300 spectrofluorometer (JASCO) and Perkin Elmer FL-6500 spectrometer in 10 mm quartz cuvettes.

Time-resolved photostability measurements were recorded with the Perkin Elmer FL-6500 spectrometer.

Photochemical setup

Photoreaction were performed by irradiation with a 40 W LED PR160L-427nm (Kessil, average intensity of PR160 series 352 mW/cm^2, measured from 1 cm distance)[a] set to full intensity. The reactions were held close to room temperature via a small fan. The reaction mixtures were set up in soda-lime glass 12 mL screw cap tubes or in 20 mL borosilicate reaction tubes and were placed in 5–7 cm distance from the light source. The absorption of the glass should be negligible at the wavelength range used.

Photostability measurements were performed by irradiating the solution in a custom-made Schlenk-cuvette with a 40 W LED PR160L-390 nm from Kessil.

NMR spectroscopy

NMR spectra were recorded on a Bruker Avance-III HD spectrometer (^{1}H NMR: 300 MHz, ^{13}C NMR: 75.5 MHz). Chemical shifts are referenced to residual solvent signals ($CDCl_3$: ^{1}H 7.26 ppm, ^{13}C 77.16 ppm) and reported in parts per million (ppm) relative to tetramethylsilane (TMS).

Laser flash photolysis

The LP980KS setup from Edinburgh Instruments equipped with an Nd:YAG laser from Litron (Nano LG 300-10) was employed for transient absorption and time-resolved emission spectroscopy. The frequency-tripled output with a wavelength of 355 nm served as the excitation source. The laser pulse duration was ≈5 ns and the pulse frequency was 10 Hz. The typical pulse energy used for transient absorption and emission studies was 20 mJ. A beam expander was used to increase the spatial overlap between laser pulse and probe lamp. A beam compressor was used to increase the laser intensity/area. Detection of transient absorption spectra occurred on an iCCD camera from Andor. Kinetic traces at

---

[a] As stated by the manufacturer.

selected wavelengths were recorded using a photomultiplier tube. The laser power was measured using a pyroelectric detector with attenuator (QE25LP-S-MB-QED-D0) from Gentec-EO combined with the integra software. The spectroscopic experiments were performed at 293 K using a cuvette holder that allows temperature control. If not stated otherwise the TA spectra were integrated over 100 ns.

77 K measurements were carried out with the LFP setup. The cuvette holder was replaced by a dewar that positions a 4 mm glass tubes within the laser beam. The singlet state and triplet state energy of Aza-H were determined at the position of the first (i.e. high-energy) emission shoulder or maximum.

Fluorescence lifetime measurements

The *mini-*τ setup from Edinburgh Instruments (time-correlated single photon counting (TCSPC) technique) equipped with a pulsed LED (EPLED-375, excitation at ≈371 nm) was employed for fluorescence lifetime measurements of Aza-H at 293 K. Stray light was removed with an integrated band-pass filter from 525 nm to 575 nm.

Preparation of deoxygenated solutions for photostability measurements

Unless noted otherwise, the solutions for photostability experiments were deoxygenated in custom-made Schlenk-cuvettes [2] by three freeze-pump-thaw cycles using argon as an inert gas, liquid N_2 for cooling, and Schlenk line vacuum (down to 0.2 mbar).

II. Quantum-mechanical computations

DFT calculations were performed with the program Orca 5.0.4[3]. The functional B3LYP and the basis set 6-311+G(d,p) were used. After each geometry optimization, vibrational frequencies were calculated. In each case, no imaginary vibration frequencies were obtained, indicating convergence on minimum structures. Additional single-point calculations were carried out with Mulliken population analyses to obtain the frontier orbitals of the singlet ground state. The geometry optimization of the triplet state was performed starting from the energy-minimized singlet ground state geometry as initial guess. The triplet state energy (adiabatic) was determined by subtracting the energy of the optimized triplet state from the energy of the optimized singlet ground state. To determine electronic excitation energies and oscillator strengths of the lowest 30 transitions, TD-DFT calculations were performed with previously geometry-optimized structures at the same level of theory. For clarity, only the transitions with an oscillator strength above 0.05 are displayed in the main part (see Figure 1B). Transitions below 400 nm, where the ground-state absorption of Aza-H influences the TA spectra, were also excluded. Additional single-point calculations were carried out to obtain the spin densities of the optimized triplet or doublet states based on Mulliken population analysis. The structures, spin densities (iso value: 0.005) and frontier orbitals were displayed using the software Avogadro1.2.0.[4] The color code for the atoms in the ball and stick representation is as follows: carbon (black), hydrogen (white), nitrogen (bue). In the depiction of spin densities, blue-colored surfaces denote positive spin densities, whereas red-colored surfaces denote negative spin densities (as in Figure 1B of the main paper). The different signs of the wave function in the depiction of the frontier orbitals are also indicated by the colors blue and red.

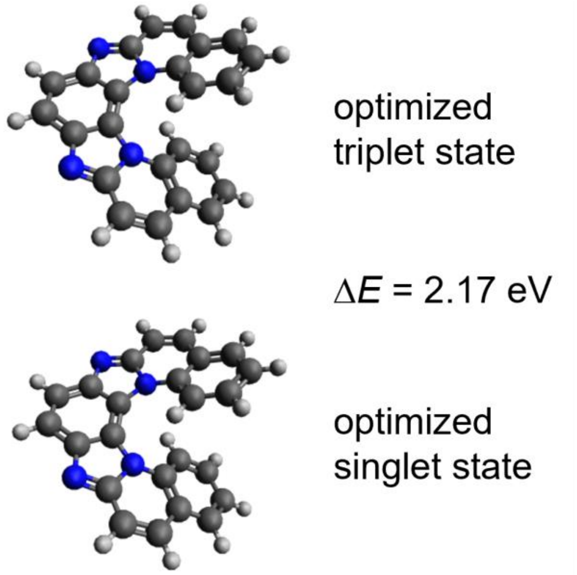

Figure S1: Left: Optimized structures of the Aza-H singlet ground state and the lowest-energy triplet state of Aza-H at B3LYP/6-311+G(d,p) level of theory, along with the respective energy difference.

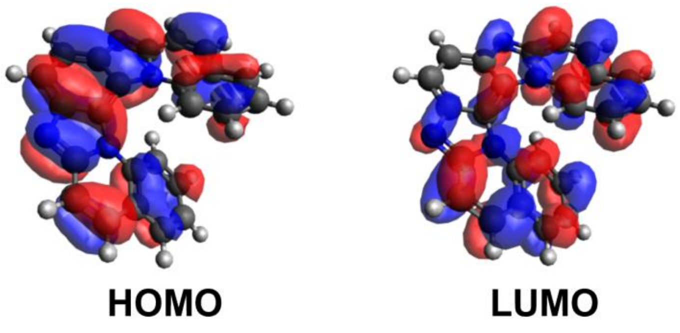

Figure S2: Frontier orbitals of the Aza-H singlet ground state.

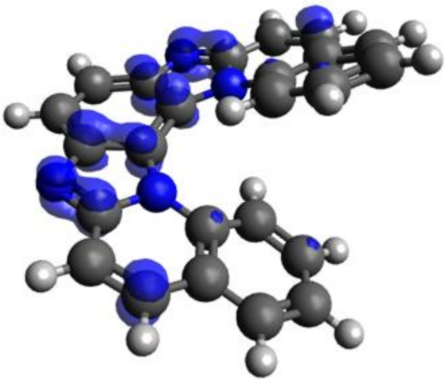

Figure S3: Structure and spin density of geometry-optimized Aza-H$^{\bullet+}$ (charge: +1, multiplicity: 2).

III. Additional mechanistic and kinetic data

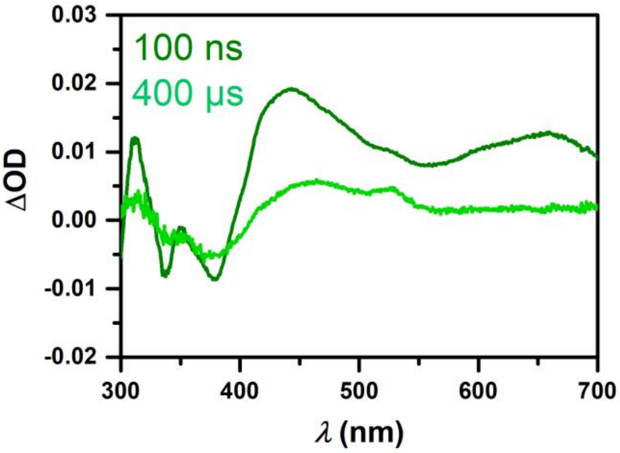

Figure S4: TA spectra of 25 µM Aza-H in Ar-saturated MeCN/H_2O (9:1) recorded 100 ns and 400 µs after 355 nm laser pulses.

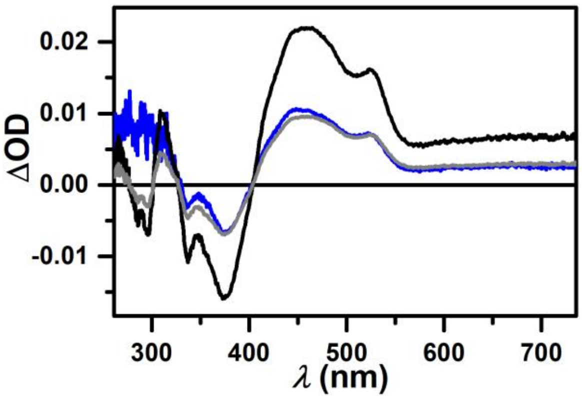

Figure S5: TA spectra in MeCN/H_2O (9:1) recorded 250 ns after 355 nm laser pulses. 25 μM Aza-H in aerated solution (black), 25 μM Aza-H with 200 mM 4CP in Ar-saturated solution (blue). The spectrum of Aza-H was obtained using optics that increase the laser intensity/area by a factor of 4, thereby resulting in higher ionization yields of Aza-H and a spectrum of higher quality accordingly. The spectrum of solely Aza-H has been scaled to fully match the spectrum with 200 mM 4CP (gray).

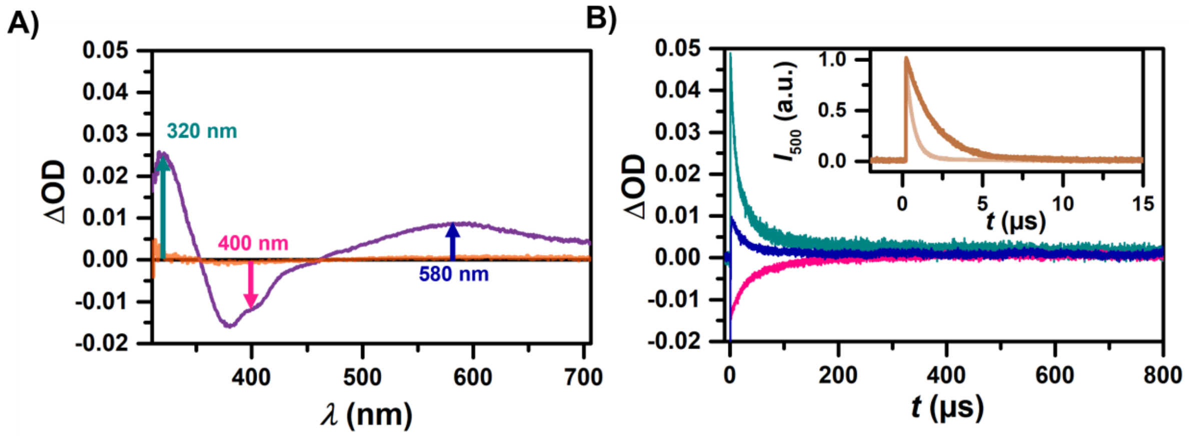

Figure S6: Mechanistic investigations of a solution with 10 μM $Ir(ppy)_3$ and 32.2 mM 4CP in deaerated MeCN with 355 nm laser pulses. A) TA spectra recorded 10 μs (purple) and 200 μs (orange) after excitation. B) Kinetic traces at 320 nm (dark green), 400 nm (pink) and 580 nm (dark blue). Inset: Kinetic emission of $Ir(ppy)_3$ in the absence and presence of 4CP.

It has been reported that, similar to Aza-H, the excited state of $Ir(ppy)_3$ can initiate the reduction of 4CP[5,6], resulting in the formation of the one-electron oxidized $Ir(ppy)_3$ and the radical anion of 4CP. LFP was used to investigate a solution containing 10 μM $Ir(ppy)_3$ and 32.2 mM 4CP in MeCN, with the aim of identifying the radical anion of 4CP. With the addition of 4CP, the phosphorescence of $Ir(ppy)_3$ is substantially quenched (Figure S6B, inset). A TA spectrum was recorded 10 μs after excitation ensuring that $Ir(ppy)_3$* is fully quenched (Figure S6A). In comparison to the literature spectrum of $Ir(ppy)_3^{\bullet+}$ [7], no detectable absorbing species is observed at the reported absorption maximum of 398 nm for $4CP^{\bullet-}$ [8]. Time-resolved measurements were conducted at 320 nm, 400 nm, and 580 nm, revealing similar decay kinetics. Following the decay of $Ir(ppy)_3^{\bullet+}$, a TA spectrum was obtained 200 μs after excitation, which showed only a baseline level (Figure S6A). Based on these results, we conclude that the 4CP radical anion exhibits weak absorption and is not detectable when reduced by either Aza-H or $Ir(ppy)_3$ using our LFP setup.

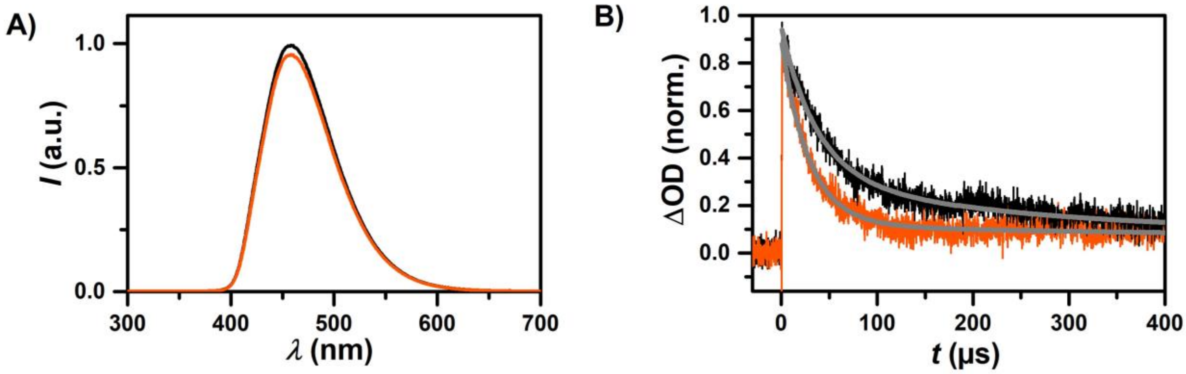

Figure S7: Quenching experiments of Aza-H with 355 nm laser pulses in the absence (black) and presence (orange) of 100 mM α-methylstyrene in deaerated MeCN/H_2O (9:1). A) Emission spectra recorded 10 ns after excitation. B) Kinetic measurements at 650 nm with corresponding triplet lifetimes.

The kinetic measurements at 650 nm were fitted with a biexponential decay function. The fit yielded two lifetimes of which the shorter is assigned to the triplet state of Aza-H. A triplet lifetime reduction from 36.2 µs to 28.6 µs is obtained by the addition α-methylstyrene. The lifetimes are slightly longer than the isolated lifetime of ^{3}Aza-H (≈28 µs, see main paper for details) due to the superimposed and non-monoexponential decay of the radical cation.

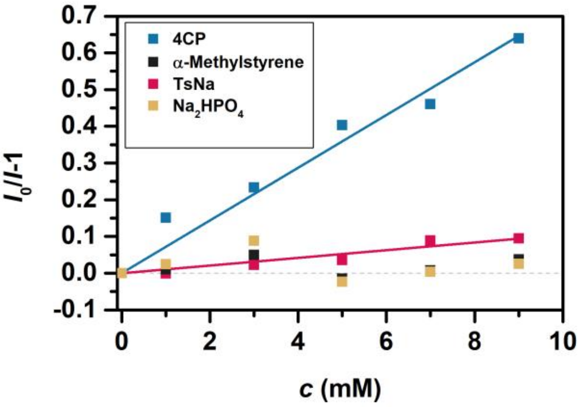

Figure S8: Stern–Volmer plots of the fluorescence quenching of Aza-H by components/additives of the 3-CR. 4CP quenches the fluorescence (K_{SV} = 71.8 M^{-1}) efficiently. TsNa shows weaker quenching (K_{SV} = 10.5 M^{-1}) and the addition of α-methylstyrene and Na_2HPO_4 shows no or only minor quenching of the singlet state.[1]

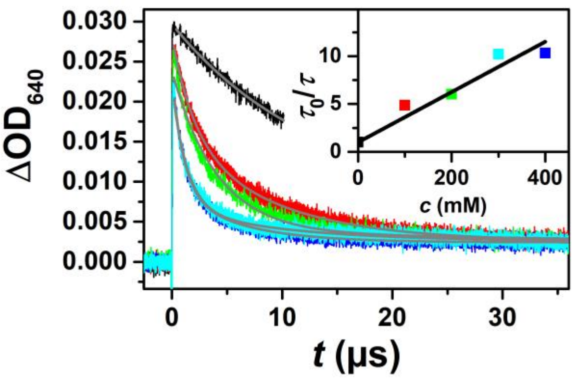

Figure S9: Triplet quenching experiments of Aza-H upon 355 nm excitation in Ar-saturated MeCN solution with increasing cinnamyl chloride concentration. Inset: corresponding Stern–Volmer plot using the lifetime of the short-lived species from the respective biexponential fit.

Moreover, the involvement of protonated states of the catalyst was investigated. Since the structure of Aza-H contains two (benz)imidazole moieties, the pK_a of the first protonation step was expected to be in a similar range (imidazole 7.05, benzimidazole 5.55) [9] for the ground state. In excited state Aza-H the protonation may occur at a different pH. The ground state and excited state pK_a values of Aza-H were determined from absorption and fluorescence spectra respectively measured at different pH values in aqueous EtOH (10%, $c = 10^{-5}$ M) (Figure S10). The absorption values at the wavelengths 230 nm and 355 nm were plotted against the pH. As two protonation sites are available in Aza-H the presence of two pK_a values was expected and is visible as two steps in the absorption plots (Figure S11). The data were fitted with [Eq. (1)], which describes the equilibrium of two interdependent pK_a values [7].

$$A_{\mathrm{total}} = \frac{A_{\mathrm{A_2}} + A_{\mathrm{A_2H}_m} 10^{m(\mathrm{p}K_2 - \mathrm{pH})} + A_{\mathrm{A_2H}_{n+m}} 10^{n \cdot \mathrm{p}K_1 + m \cdot \mathrm{p}K_2 - (n+m)\mathrm{pH}}}{1 + 10^{m(\mathrm{p}K_2 - \mathrm{pH})} + 10^{n \cdot \mathrm{p}K_1 + m \cdot \mathrm{p}K_2 - (n+m)\mathrm{pH}}}$$

[Eq. (1)]

Prior to analysis, the fluorescence spectra were corrected with the factor $A_{280\mathrm{nm},7.825} \cdot A_{280\mathrm{nm,pH}}^{-1}$ to account for the pH dependence of the absorbance at the excitation wavelength (280 nm). The emission intensities at the wavelengths 460 nm and 540 nm were plotted against the pH and fitted with (Eq. 1), substituting the absorbance for the fluorescence intensity, yielding pK_1 and pK_2 of the excited state Aza-H*****. The ground state pK_a values calculated from the absorbance data at 230 nm and 355 nm are in agreement within the range of the standard error, while the excited state values obtained from the fluorescence intensities at 460 nm and 540 nm differ by 0.31 for pK_1 and 0.40 for pK_2, taking their errors into account. Nevertheless, a shift in acidity of about two orders of magnitude for pK_1 and about two and a half orders of magnitude for pK_2 can be observed upon excitation of Aza-H, with the excited Aza-H* being much more readily protonated.

The acidity of the catalyst in the ground state is higher (pK_a = 3.70) than for benzimidazole and imidazole, while a protonation of the excited state is already observed under slightly acidic conditions (pH < 6) in the solvent system used. However, the addition of a base (Na_2HPO_4) to the conducted 3-CR, resulting in an increased pH, and the low water content of 10% make the contribution of protonated forms of the catalyst unlikely. As was shown previously [1] the addition of an acid in a similar reaction does not deactivate the catalyst, making it a reasonable assumption that the protonated form also exhibits catalytic activity.

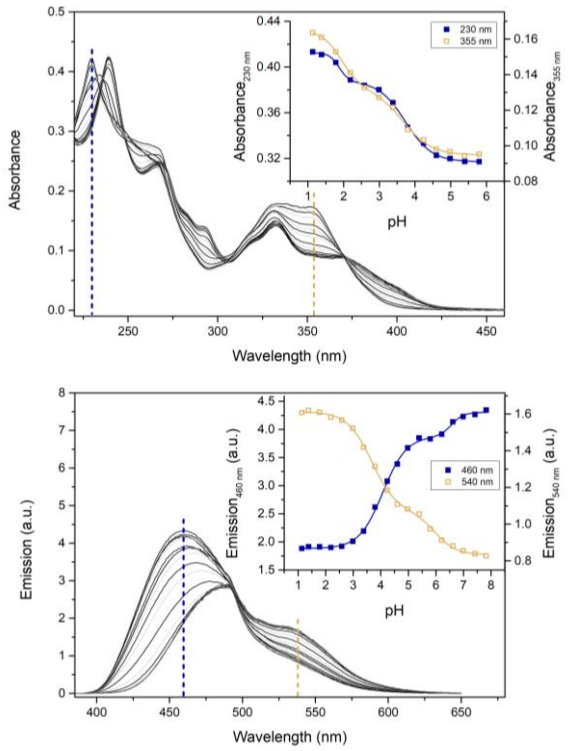

Figure S10: pH dependent absorbance and emission of Aza-H. Insets: Plot of absorption and emission against pH at specific wavelengths and curve fits with (Eq. 1).

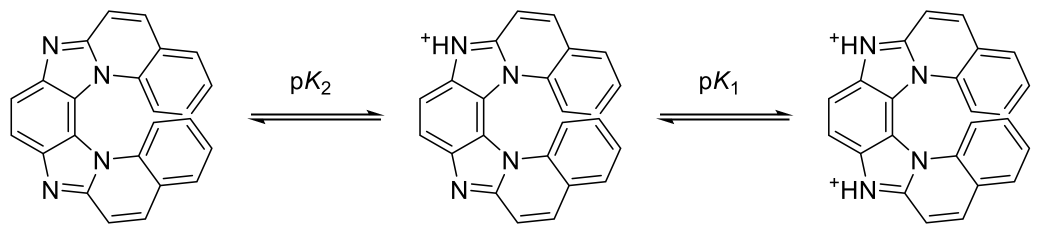

Aza-H **HAza-H⁺** **H_2Aza-H^{2+}**

Figure S11: Molecular structures of Aza-H and first and second protonation step of Aza-H.

Table 1**:** pK_a values of Aza-H in ground and exited state.

	pK_1	pK_2
**Ground State**		
230 nm	1.87±0.05	3.73±0.04
355 nm	1.93±0.07	3.67±0.11
**Excited State**		
460 nm	4.07±0.03	6.57±0.09
540 nm	3.67±0.06	5.99±0.09

IV. Photostability measurements

The photostability of Aza-H was compared to riboflavin, $[Ru(bpy)_3](PF_6)_2$ and 4CzIPN. A 390 nm LED from Kessil was selected as the excitation light source, since all four chromophores readily absorb at this wavelength. The optical density of all four photocatalysts was standardized to 0.1 at 390 nm. Emission spectra were taken before and after the photostability measurement to detect any emissive species generated during the experiment. However, no new emissive species were detected, allowing us to relate the emission intensity to the concentration. The relative degradation was estimated by fitting a linear function to the first 10 minutes (2 minutes for riboflavin due to fast decomposition) of the experiment.

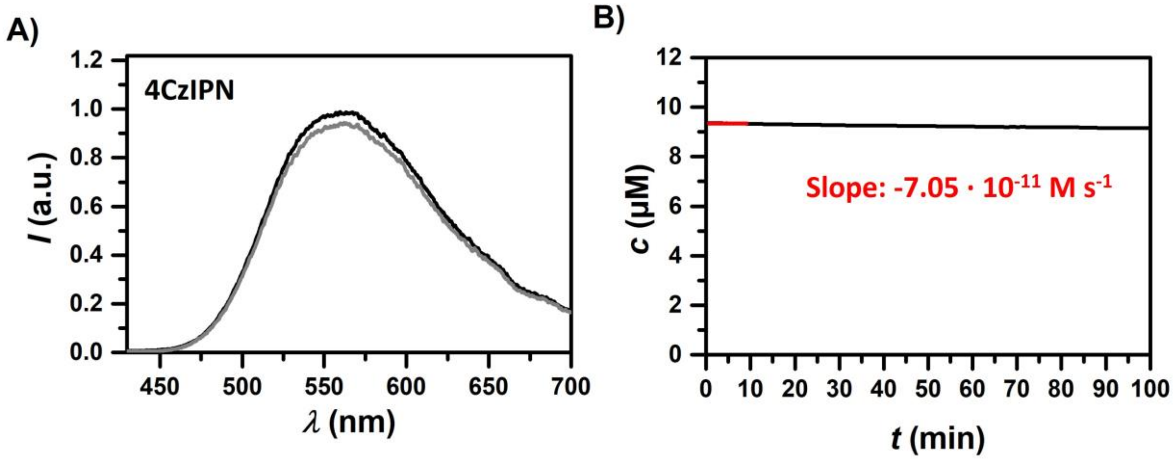

Figure S12: Photostability measurement of 9.3 µM 4CzIPN in deaerated MeCN. A 390 nm LED from Kessil set to 50% intensity (distance 6 cm) was used as the excitation light source. A) Emission spectrum recorded before (black) and after (gray) the measurement. B) Concentration (derived from the emission intensity recorded at 550 nm) of 4CzIPN plotted against the irradiation time. The first 10 min were used to determine the slope.

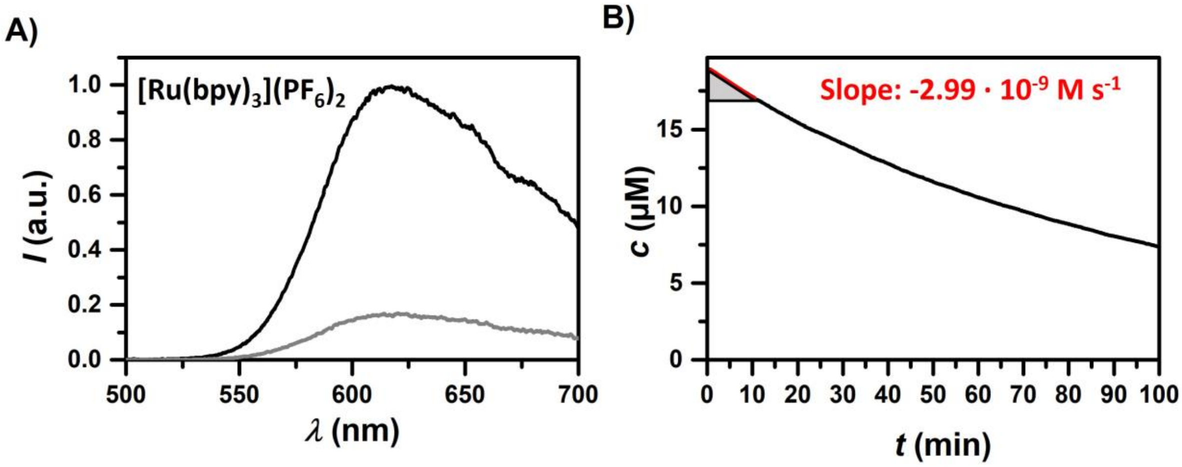

Figure S13: Photostability measurement of 19.0 µM $[Ru(bpy)_3](PF_6)_2$ in deaerated MeCN. A 390 nm LED from Kessil set to 50% intensity (distance 6 cm) was used as the excitation light source. A) Emission spectrum recorded before (black) and after (gray) the measurement. B) Concentration (derived from the emission intensity recorded at 600 nm) of $[Ru(bpy)_3](PF_6)_2$ plotted against the irradiation time. The first 10 min were used to determine the slope.

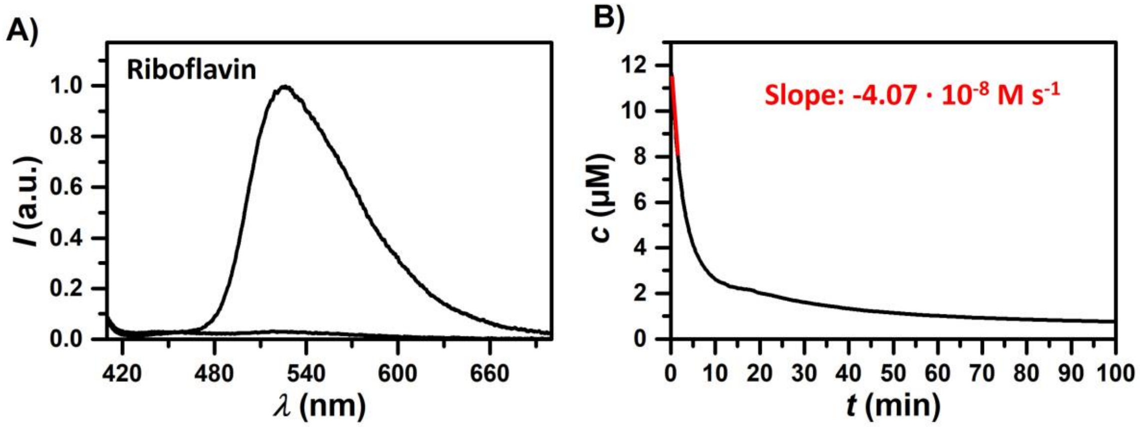

Figure S14: Photostability measurement of 11.2 µM riboflavin in deaerated H_2O. A 390 nm LED from Kessil set to 50% intensity (distance 6 cm) was used as the excitation light source. A) Emission spectrum recorded before (black) and after (gray) the measurement. B) Concentration of riboflavin plotted against the irradiation time. The first 2 min were used to determine the slope.

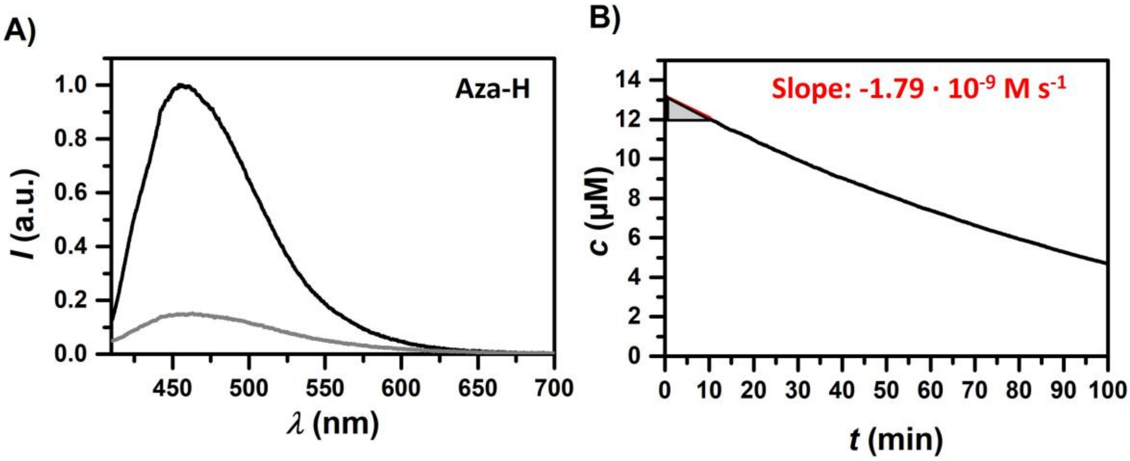

Figure S15: Photostability measurement of 13.2 µM Aza-H in deaerated MeCN. A 390 nm LED from Kessil set to 50% intensity (distance 6 cm) was used as the excitation light source. A) Emission spectrum recorded before (black) and after (gray) the measurement. B) Concentration (derived from the emission intensity recorded at 455 nm) of Aza-H plotted against the irradiation time. The first 10 min were used to determine the slope.

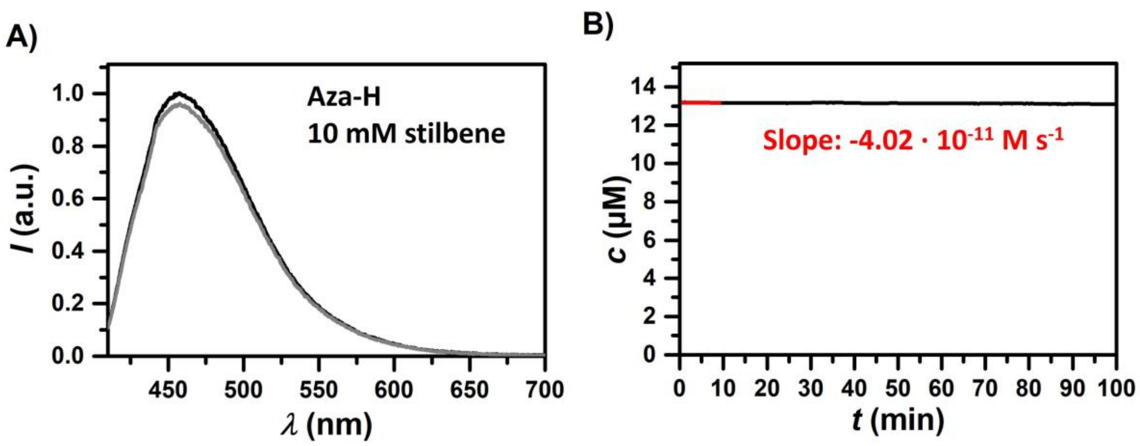

Figure S16: Photostability measurement of 13.2 µM Aza-H and 10 mM (*E*)-stilbene in deaerated MeCN. A 390 nm LED from Kessil set to 50% intensity (distance 6 cm) was used as the excitation light source. A) Emission spectrum recorded before (black) and after (gray) the measurement. B) Concentration (derived from the emission intensity recorded at 455 nm) of Aza-H plotted against the irradiation time. The first 10 min were used to determine the slope.

The addition of 10 mM (*E*)-Stilbene ensures that the triplet-excited Aza-H is rapidly quenched. We observe that the inherent photostability of Aza-H increases by a factor of 44. Hence, we attribute the comparatively fast decomposition of (unquenched) Aza-H to the long-lived triplet state.

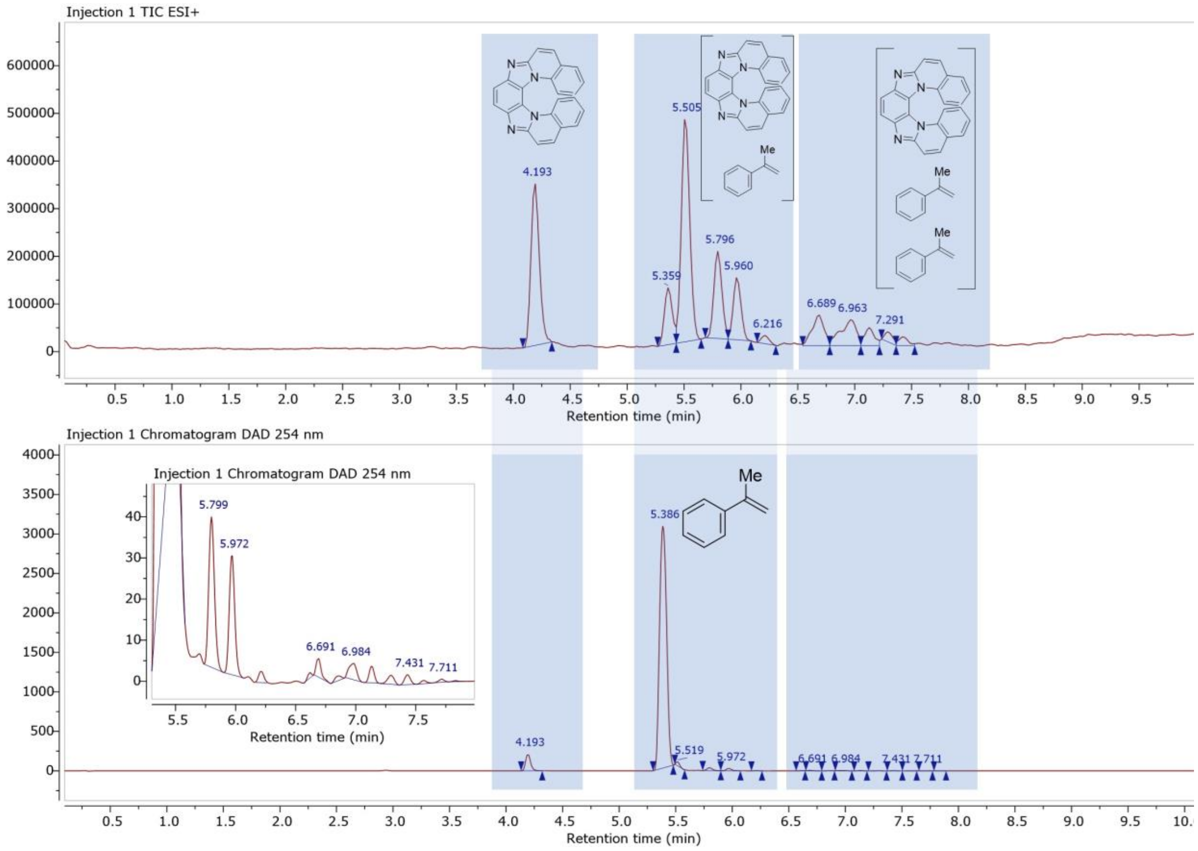

Figure S17: Total ion count and UV-absorption traces from LC-MS measurements of irradiated solution of Aza-H and α-methylstyrene. Five peaks are found which correlate to mono-addition of the styrene and ten peaks correlate to double-addition of the styrene to Aza-H.

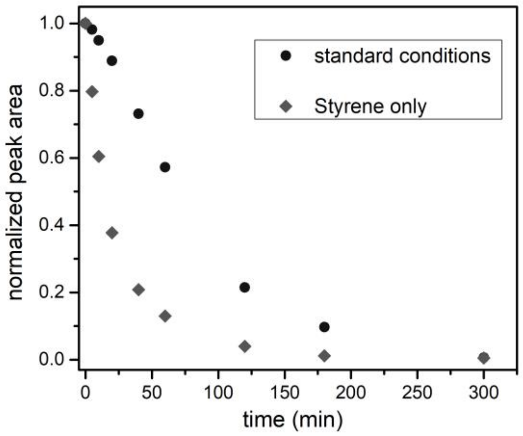

Figure S18: Reaction course plots of the normalized peak area of Aza-H under standard conditions and with styrene only. Standard conditions: To the 12 mL tube equipped with a magnetic stir bar cyanopyridine (0.15 mmol, 1.0 equiv), sodium sulfinate (0.1 mmol, 2.0 equiv), $Na_2HPO_4{\cdot}H_2O$ (0.3 mmol, 53.4 mg, 2.0 equiv) and azahelicene catalyst 9 (7.5 µmol, 2.7 mg, 0.05 equiv) were added and sealed with a septum. The vial was then evacuated and flushed with nitrogen three times. Styrene (0.3 mmol, 2.0 equiv) and freshly degassed acetonitrile/H_2O (9:1, 8 mL) were added via syringe [1].

V. Additional information for the isomerization of stilbene and cinnamyl chloride

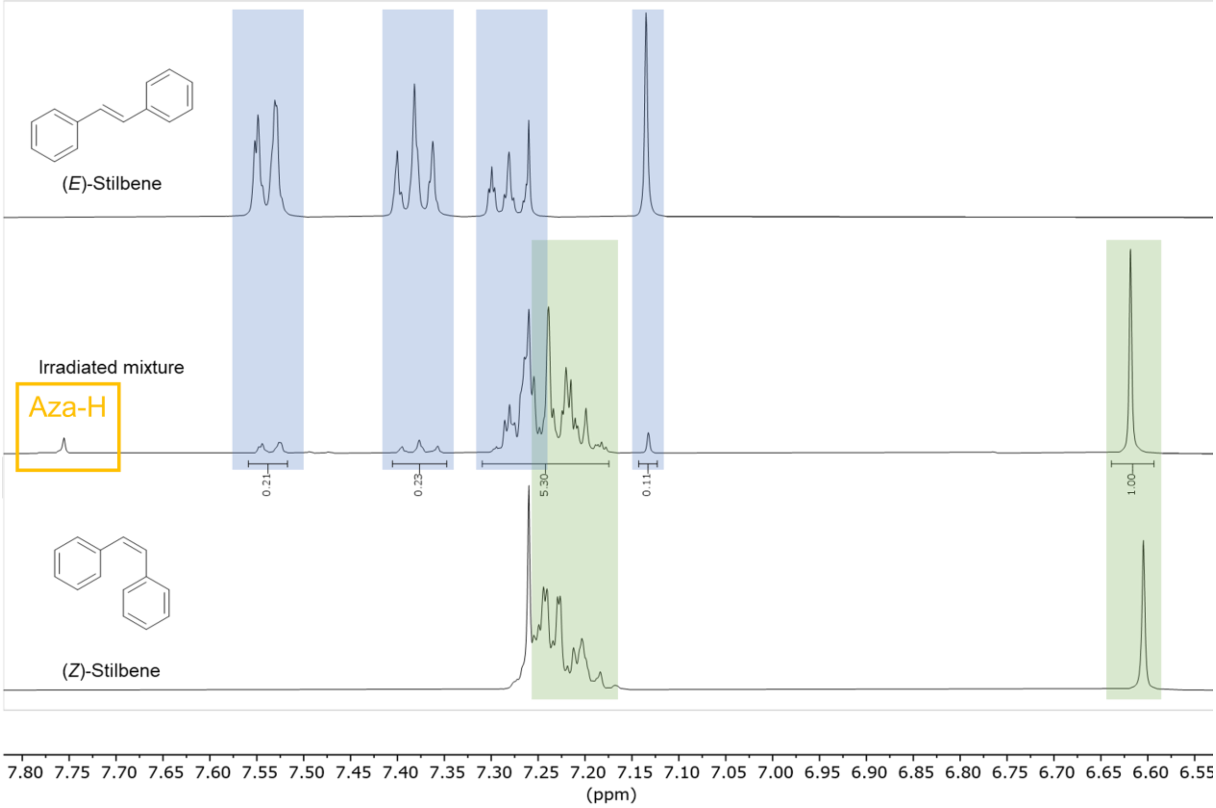

Figure S19: Sample NMR spectra of (*E*)-stilbene (top), the irradiated mixture (1 h, 427 nm) with Aza-H (middle) and (*Z*)-stilbene (bottom). Conditions: (*E*)-Stilbene 50 mM + Aza-H 1 mM in degassed MeCN.

The chemical shifts of the ethylene protons of the irradiated mixture agree with the control measurements of the commercially obtained isomers (7.13 ppm for (*E*)-stilbene and 6.61 ppm for (*Z*)-stilbene). The ratio of E/Z was determined by the signal intensity of (*E*)-stilbene at 7.13 ppm and (*Z*)-stilbene at 6.61 ppm. The signal at 7.75 ppm is assigned to the photocatalyst Aza-H. GC analysis confirmed the *E*/*Z*-ratios obtained from the NMR spectra and revealed no side product formation after the reaction. For the reaction course plot (main article Figure 5D), samples were taken from an irradiated mixture of stilbene (single isomer, 50 mM) and Aza-H (1 mM) in MeCN after times *t* and subjected to GC analysis. Integration of the FID trace was used to determine the *E*/*Z*-ratios.

For the TON experiments, a MeCN solution containing Aza-H (0.1 mM, 0.07 mol %) and nearly saturated with (*E*)-stilbene (0.14 M) was irradiated for 14 h (427 nm). GC analysis gave an *E*/*Z*-ratio of 17:83 corresponding to a TON of 1160. An even higher TON was achieved, when a suspension of (*E*)-stilbene ("1 M") and Aza-H (0.1 mM, 0.01 mol %) in MeCN was irradiated. After 100 h, an *E*/*Z*-ratio of 56:44 corresponding to a TON of 4400 was observed. The generated liquid *Z*-isomer is more soluble in the solvent MeCN and remains in solution, allowing more *E*-isomer to dissolve and isomerize.

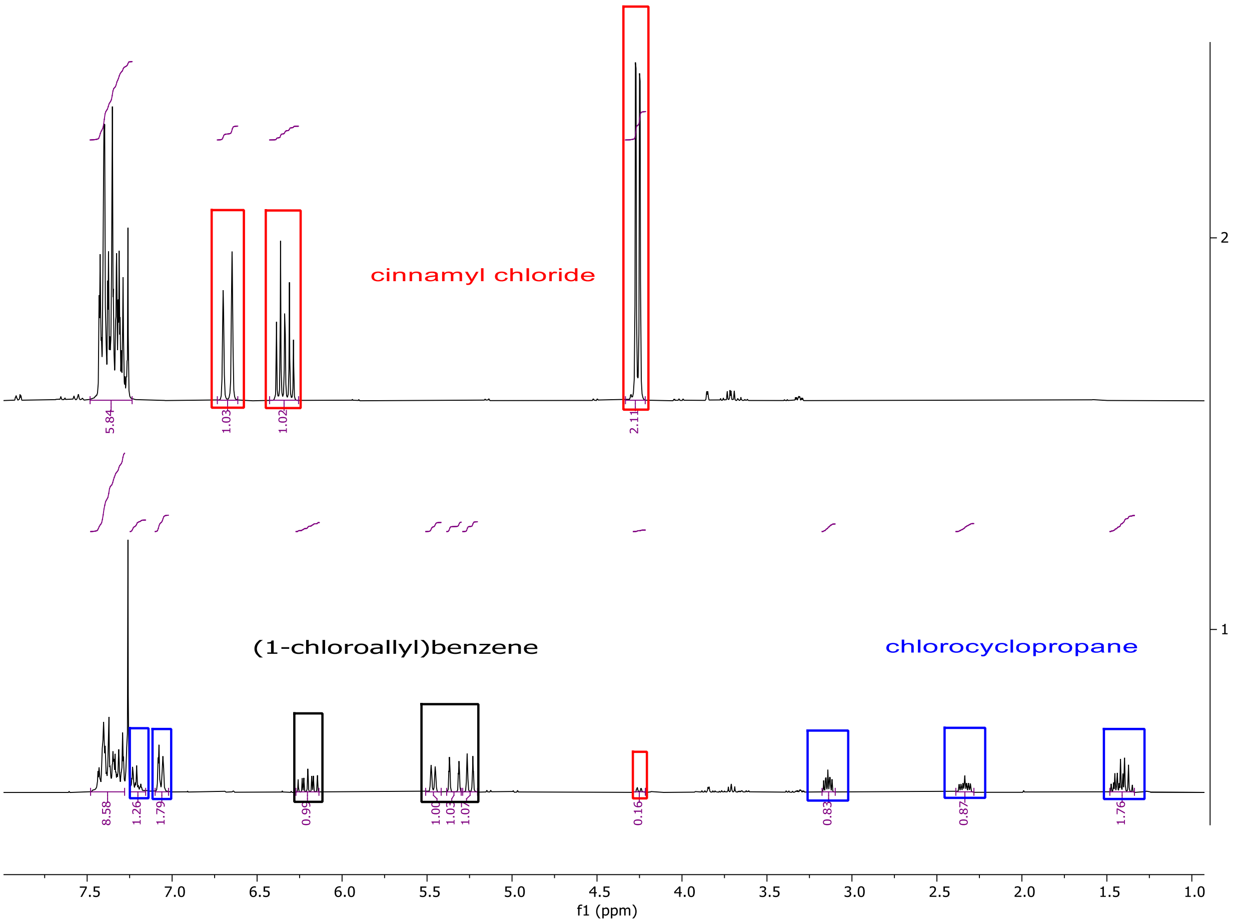

Figure 20: NMR spectra of the cinnamyl chloride cyclization reaction before and after irradiation in $CDCl_3$. The solution of cinnamyl chloride (0.2 M) and Aza-H (2 mM) in MeCN was irradiated for 2 h (427 nm).

The NMR peaks of the chlorocyclopropane and the (1-chloroallyl)benzene are in accordance with the literature[10]. The yield of the chlorocyclopropane and (1-chloroallyl)benzene were determined by the relative ratios of the isolated signals. (3.17–3.12 ppm for the chlorocyclopropane (2H), 5.48 ppm for (1-chloroallyl)benzene (2H) and 4.25 ppm for cinnamyl chloride (2H)). No side products were observed by NMR or GC.

VI. References

(1) Rocker, J.; Opatz, T. *ACS Org. Inorg. Au* **2022**, *2*, 415–421. doi:10.1021/acsorginorgau.2c00022
(2) Zähringer, T. J. B.; Bertrams, M.-S.; Kerzig, C. *J. Mater. Chem. C* **2022**, *10*, 4568–4573. doi:10.1039/D1TC04782E
(3) Neese, F. *WIREs Comput. Mol. Sci.* **2012**, *2*, 73–78. doi:10.1002/wcms.81
(4) Hanwell, M. D.; Curtis, D. E.; Lonie, D. C.; Vandermeersch, T.; Zurek, E.; Hutchison, G. R. *J. Cheminformatics* **2012**, *4*, 17. doi:10.1186/1758-2946-4-17
(5) Vorob'ev, Y. *Chem. Heterocycl. Compd.* **2019**, *55*, 90–92
(6) Lipp, B.; Kammer, L. M.; Kücükdisli, M.; Luque, A.; Kühlborn, J.; Pusch, S.; Matulevičiūtė, G.; Schollmeyer, D.; Šačkus, A.; Opatz, T. *Chem. Eur. J.* **2019**, *25*, 8965–8969. doi:10.1002/chem.201901175
(7) Zhao, H.; Simpson, P. V.; Barlow, A.; Moxey, G. J.; Morshedi, M.; Roy, N.; Philip, R.; Zhang, C.; Cifuentes, M. P.; Humphrey, M. G. *Chem. Eur. J.* **2015**, *21*, 11843–11854. doi:10.1002/chem.201500951
(8) Kalyanaraman, V.; Rao, C. N. R.; George, M. V. *J. Chem. Soc. B Phys. Org.* **1971**, 2406. doi:10.1039/j29710002406
(9) Walba, H.; Isensee, R. W. *J. Org. Chem.* **1961**, *26*, 2789–2791. doi:10.1021/jo01066a039
(10) Xu, B.; Troian-Gautier, L.; Dykstra, R.; Martin, R. T.; Gutierrez, O.; Tambar, U. K. *J. Am. Chem. Soc.* **2020**, *142*, 6206–6215. doi:10.1021/jacs.0c00147
